# Diaschisis as Cerebello-Cortical Loop Dysfunction in Acute Ischemic Stroke: A Network Framework for Outcome Variability

**DOI:** 10.3390/brainsci16060594

**Published:** 2026-05-30

**Authors:** Nannan Sheng, Qi Jia, Gilles Naeije

**Affiliations:** Neurology Department, Université Libre de Bruxelles, Cliniques Universitaires de Bruxelles, Hôpital Erasme, 1070 Bruxelles, Belgium; nannan.sheng@ulb.be (N.S.); qi.jia@ulb.be (Q.J.)

**Keywords:** ischemic stroke, diaschisis, crossed cerebellar diaschisis, thalamic diaschisis, neuroimaging, perfusion imaging, PET, SPECT, CT perfusion, functional connectivity, stroke outcome, brain networks

## Abstract

**Highlights:**

This conceptual review reframes diaschisis after ischemic stroke as a structured dysfunction of cerebello-cortical networks rather than a nonspecific remote effect. It highlights how this network-based perspective can improve outcome prediction and inform targeted rehabilitation and neuromodulation strategies.

**What are the main findings?**

- Diaschisis after ischemic stroke reflects structured dysfunction within cerebello-cortical and thalamo-cortical loops rather than a nonspecific remote effect.
- Multimodal evidence (perfusion, PET/SPECT, and functional connectivity) converges to support a network-based model explaining inter-individual variability in motor and cognitive outcomes.

**What are the implications of the main findings?**

- A network framework of diaschisis provides a mechanistic basis for improving prognostic models beyond lesion location and volume alone.
- Targeting cerebello-cortical circuits may open new avenues for personalized rehabilitation and neuromodulation strategies in stroke recovery.

**Abstract:**

Clinical outcomes after acute ischemic stroke remain highly heterogeneous, even among patients with comparable lesion characteristics and successful reperfusion, challenging traditional lesion-based models. Increasing evidence suggests that stroke should be conceptualized as a disorder of distributed brain networks, yet the mechanisms linking focal ischemia to large-scale dysfunction remain incompletely understood. In this review, we propose that diaschisis constitutes a central physiological mechanism underlying this transition from focal injury to network-level impairment. Building on advances in functional imaging, connectomics, and cerebellar physiology, we propose that diaschisis may be conceptualized, at least in part, as a disruption of cerebello-cortical loop dynamics rather than solely a nonspecific remote effect. These closed, polysynaptic circuits linking cortex, cerebellum, and thalamus support the integration of motor and cognitive processes and are particularly vulnerable to perturbation. Focal ischemia may therefore induce a cascade of dysfunction that propagates across these loops, leading to widespread impairment despite limited structural damage. Within this framework, outcome variability emerges from the interaction of three key factors: lesion characteristics, brain reserve and network vulnerability, and the extent of diaschisis. We further highlight that functional suppression of cerebellar output, even in the absence of structural degeneration, may play a critical role in mediating network dysfunction. This circuit-based perspective provides a mechanistic explanation for inter-individual variability in stroke outcomes and shifts the focus from lesion localization to network dynamics. Understanding diaschisis as a potential manifestation of cerebello-cortical loop dysfunction opens new avenues for prognosis and therapeutic intervention, emphasizing the potential of targeting network-level restoration to improve recovery after stroke.

## 1. Introduction—The Limits of Lesion-Based Models

Clinical outcomes after acute ischemic stroke remain highly heterogeneous, even in the era of effective reperfusion therapies. Patients with comparable infarct volumes, lesion locations, and recanalization status frequently exhibit markedly different trajectories, ranging from near-complete recovery to persistent motor and cognitive disability. This variability challenges the traditional lesion-based framework, in which clinical deficits are assumed to be primarily determined by the size and anatomical location of the infarct.

Although lesion characteristics and baseline neurological severity, commonly assessed using the National Institutes of Health Stroke Scale (NIHSS), are robust predictors of outcome, they explain only part of the observed variance. Large randomized trials and pooled analyses of endovascular thrombectomy have consistently shown that substantial inter-individual variability persists even after accounting for age, treatment delay, and recanalization success [1,2,3]. Moreover, the relationship between infarct volume and functional outcome is not deterministic, particularly among patients with successful reperfusion, suggesting that structural damage alone does not fully capture the mechanisms underlying clinical evolution [4].

These limitations have prompted a shift toward conceptualizing stroke as a disorder of distributed brain systems rather than a purely focal injury. Early descriptions of diaschisis highlighted that focal lesions can induce functional depression in remote but anatomically connected regions [5]. More recent work using functional neuroimaging has extended this view, demonstrating that disruption of large-scale brain networks contributes substantially to post-stroke deficits. In particular, alterations in functional connectivity have been shown to predict impairments across multiple behavioral domains beyond what can be explained by lesion location alone [6,7,8].

However, while these network-based approaches have advanced our understanding of stroke pathophysiology, a fundamental question remains unresolved: why do patients with apparently similar lesions exhibit such divergent outcomes? In other words, what determines the extent to which a focal ischemic insult translates into widespread functional impairment? Addressing this question requires moving beyond lesion-centric models and considering both the intrinsic properties of brain networks and the mechanisms through which focal injury disrupts their function. In the following sections, we examine how inter-individual differences in network organization and vulnerability may contribute to outcome variability, before exploring the role of diaschisis as a key physiological mechanism linking focal ischemia to distributed brain dysfunction.

## 2. Brain Reserve and Network Vulnerability

The marked variability in clinical outcomes following acute ischemic stroke suggests that the impact of a focal lesion depends not only on its anatomical characteristics but also on the pre-existing organization and integrity of brain networks. This concept is captured by the framework of brain reserve, which refers to the capacity of the brain to tolerate structural injury while maintaining function. Brain reserve is shaped by multiple factors, including structural connectivity, synaptic density, network efficiency, and lifetime exposures, and has emerged as a key determinant of resilience to neurological insults [9]. In the context of stroke, brain reserve can be understood as the ability of distributed networks to compensate for focal disruption through redundancy, reorganization, and adaptive plasticity. Individuals with more efficient or robust network architectures may be better able to maintain functional integration despite localized damage, whereas others may exhibit disproportionate impairment due to disruption of critical network hubs or pathways. This perspective is supported by studies demonstrating that pre-existing brain network organization influences both acute deficits and recovery trajectories, independently of lesion size [8].

A key structural correlate of reduced brain reserve is brain atrophy, which reflects cumulative neurodegenerative, vascular, and aging-related processes. Increasing evidence indicates that global and regional brain atrophy significantly modulates stroke outcomes. Patients with greater pre-existing atrophy tend to have worse functional recovery, even after successful reperfusion, suggesting that reduced structural reserve limits the capacity for network compensation [10,11,12]. Importantly, this effect appears to be independent of infarct volume, reinforcing the idea that baseline brain integrity is a critical determinant of outcome. Beyond global atrophy, regional patterns of structural vulnerability may further shape network resilience. In particular, atrophy affecting key nodes within large-scale networks, such as fronto-parietal, default mode, or subcortical systems, may reduce the efficiency of information processing and limit the brain’s capacity to redistribute function after injury [13]. From a network perspective, atrophy can thus be viewed as a reduction in the redundancy and robustness of connectivity, rendering the system more susceptible to disruption by focal lesions [14].

Within this framework, the concept of network vulnerability emerges as a complementary notion to brain reserve. Rather than focusing solely on the capacity to compensate, network vulnerability emphasizes the susceptibility of specific circuits to failure when challenged by acute perturbations. This vulnerability is likely determined by a combination of factors, including network topology, metabolic demand, and dependence on long-range connections. Highly integrated and polysynaptic systems may be particularly at risk, as disruption at one node can propagate across the network and lead to widespread dysfunction [13,15]. These considerations suggest that stroke outcome variability arises from an interaction between the structural state of the brain prior to the insult and the functional consequences of the lesion within distributed networks. While brain reserve and atrophy define the baseline capacity and vulnerability of these networks, they do not fully explain how focal ischemia leads to remote and system-level dysfunction. In particular, the mechanisms through which acute hemodynamic disturbances translate into large-scale alterations of network activity remain insufficiently understood [5,8].

In this context, diaschisis represents a plausible physiological process linking focal injury to distributed network dysfunction. By inducing remote suppression of activity in structurally intact but functionally connected regions, diaschisis may reveal the latent vulnerability of brain networks and interact with pre-existing structural reserve to shape clinical outcomes. The following section examines this phenomenon in detail and explores its role as a key mediator between focal ischemia and large-scale network disruption.

## 3. Diaschisis—A Missing Physiological Link

While brain reserve and network vulnerability provide a framework to understand inter-individual differences in resilience to stroke, they do not fully explain how a focal ischemic lesion propagates to produce widespread dysfunction across anatomically intact regions. A key missing element is the physiological mechanism by which localized hypoperfusion translates into distributed network impairment. In this context, the concept of diaschisis offers a critical link between focal injury and large-scale brain dysfunction.

Originally described by von Monakow in the early 20th century, diaschisis refers to a loss or depression of function in brain regions that are anatomically intact but connected to a damaged area [5]. Although initially conceptualized in metabolic and electrophysiological terms, modern neuroimaging has provided robust evidence that diaschisis is a common and clinically relevant phenomenon in acute ischemic stroke. Functional MRI and PET studies have consistently demonstrated reduced activity and altered connectivity in remote regions following focal lesions, supporting the idea that stroke disrupts distributed brain systems rather than isolated anatomical loci [16]. Importantly, the term diaschisis encompasses multiple levels of organization that should not be considered mechanistically interchangeable. These include: (i) metabolic or perfusion diaschisis characterized by remote hemodynamic suppression; (ii) functional connectivity alterations affecting large-scale network synchronization; and (iii) physiological neuronal dysfunction involving altered firing dynamics and synaptic activity [5,14,17]. Although these dimensions may interact, they likely represent distinct but related expressions of distributed network perturbation.

Among the different forms of diaschisis, crossed cerebellar diaschisis (CCD) has been one of the most extensively studied. CCD refers to reduced metabolism and perfusion in the cerebellar hemisphere contralateral to a supratentorial lesion and is thought to arise from disruption of cortico-ponto-cerebellar pathways. Early PET studies demonstrated that CCD correlates with both lesion characteristics and clinical deficits [18], and more recent work has shown that its persistence is associated with poorer functional outcomes [19]. These findings highlight that diaschisis is not merely an epiphenomenon but may have direct clinical relevance.

Beyond CCD, growing evidence indicates that diaschisis involves multiple interconnected regions, including the thalamus and distributed cortical areas, reflecting the organization of large-scale brain networks. Advances in perfusion imaging have further expanded this view by enabling the quantification of hemodynamic alterations across the whole brain in the acute phase of stroke. These studies demonstrate that diaschisis is highly prevalent and often extends beyond single remote regions, involving multiple nodes within interconnected systems. Importantly, the presence and extent of diaschisis appear to be driven not by infarct core volume but by the extent and severity of hypoperfusion, suggesting that acute hemodynamic stress plays a central role in shaping distributed dysfunction.

From a mechanistic perspective, diaschisis can be understood as a consequence of disrupted synaptic input and reduced neuronal activity within connected regions. Loss of afferent drive from the lesioned area leads to decreased neuronal activity, altered firing patterns, and reduced metabolic demand in downstream structures. In crossed cerebellar diaschisis, this phenomenon has been directly demonstrated using positron emission tomography, showing reduced cerebellar blood flow and oxygen metabolism contralateral to supratentorial lesions, consistent with functional deafferentation through interruption of cortico-ponto-cerebellar pathways [20]. In this sense, diaschisis represents a state of functional disconnection, in which structurally intact regions become transiently or persistently “offline” due to network-level perturbations.

Importantly, diaschisis is not uniform across the brain but appears to preferentially affect polysynaptic, highly integrated circuits, where disruption at one node can propagate across multiple levels of the network. This property suggests that diaschisis reflects the intrinsic organization of brain systems, with certain circuits being particularly vulnerable to remote effects of focal injury. In this framework, diaschisis can be viewed as the physiological expression of network failure, revealing the dependence of distributed brain function on intact connectivity and sustained synaptic activity. These findings position diaschisis as a key mechanism linking focal ischemic injury to distributed brain dysfunction. By mediating the impact of hypoperfusion across anatomically connected regions, diaschisis provides a plausible explanation for why similar lesions can produce markedly different clinical outcomes, depending on the extent and pattern of network involvement. However, despite this growing body of evidence, the specific circuits through which diaschisis translates into behavioral impairment remain incompletely characterized. In particular, identifying the network architectures most susceptible to diaschisis, and understanding how their disruption impacts motor and cognitive function, represents a critical next step. In the following sections, we explore the hypothesis that cerebello-cortical loops constitute a central substrate of diaschisis, providing a structured and physiologically grounded framework to link remote dysfunction with clinical deficits.

## 4. The Cerebellum as a Central Hub of Distributed Brain Function

The cerebellum is increasingly recognized as a key node in distributed brain systems, extending far beyond its classical role in motor coordination. Clinical and neuroimaging studies have demonstrated that cerebellar dysfunction contributes not only to motor impairment but also to cognitive and affective deficits, a concept formalized in the description of the cerebellar cognitive affective syndrome [21]. In the context of stroke, these observations are particularly relevant, as remote cerebellar dysfunction—most notably crossed cerebellar diaschisis—has been repeatedly associated with both motor and functional outcomes [19]. This functional diversity is supported by the organization of the cerebellum into topographically structured cerebello-cortical loops. Anatomical tracing and human neuroimaging studies have established that the cerebellum forms closed, polysynaptic circuits linking the cerebral cortex to the cerebellum via the pontine nuclei, and returning through the deep cerebellar nuclei and thalamus back to cortical regions [22,23]. These loops are organized in parallel channels connecting distinct cerebellar territories with sensorimotor, associative, and limbic cortical areas, embedding the cerebellum within large-scale functional networks that support behavior. Within this organization, the posterior cerebellum—particularly lobule VI and Crus I—occupies a critical position at the interface between motor and cognitive systems. Functional neuroimaging and meta-analytic work have shown that these regions are consistently engaged across tasks involving executive control, working memory, and attention, while maintaining connectivity with motor-related networks [24,25]. This dual functional embedding suggests that disruption of these territories may have widespread consequences across domains, particularly in conditions where network integrity is compromised. The cerebellum exerts its influence on distributed brain function primarily through the cerebello-thalamo-cortical pathway, which constitutes a major route for communication between cerebellar output and the cerebral cortex. Structural and functional connectivity studies have demonstrated that cerebellar projections to the thalamus and onward to cortical association areas form a key substrate for integrating motor, cognitive, and affective processes [26,27]. In stroke, this pathway is of particular importance, as disruption of cortico-ponto-cerebellar or cerebello-thalamo-cortical connections provides a mechanistic basis for crossed cerebellar diaschisis and its downstream effects on behavior. Beyond cortical networks, the cerebellum is also functionally linked to limbic and hippocampal systems, further reinforcing its role in higher-order processes. Functional connectivity studies have demonstrated interactions between posterior cerebellar regions and medial temporal structures, suggesting a contribution to memory and affective regulation [23]. These distributed connections provide a plausible substrate through which cerebellar dysfunction may contribute to multidomain deficits observed after stroke. Recent large-scale imaging studies further support the integrative role of the cerebellum in linking motor and cognitive performance. In a population-based NeuroImage study, a cerebellar structural covariance network centered on lobule VI and Crus I was found to be associated with both physical and cognitive impairment, with effects mediated by distributed regions including the thalamus and hippocampus. These findings underscore that cerebellar networks do not operate in isolation but are embedded within broader systems supporting complex behavior. These data position the cerebellum as a central hub within distributed brain networks, rather than a peripheral motor structure. Its organization into cerebello-cortical loops, its strategic localization at the motor–cognitive interface, and its strong connections with thalamic, cortical, and limbic systems make it particularly susceptible to remote effects of focal brain injury. In the context of stroke, this architecture provides a compelling framework through which diaschisis, especially when involving cerebellar circuits, may translate into widespread and multidomain functional impairment. Although the present review emphasizes cerebello-thalamo-cortical systems, diaschisis likely involves dysfunction across multiple interacting large-scale networks. Stroke-related deficits have also been associated with disruption of frontoparietal control systems, salience and default mode networks, cortico-striatal loops, and limbic pathways [28,29,30]. We focus on cerebellar loops because they provide a particularly well-characterized polysynaptic model linking perfusion abnormalities, physiological modulation, and multidomain behavioral dysfunction. However, cerebellar circuits should be considered as one potentially important substrate within a broader framework of distributed network vulnerability.

## 5. A Physiological Perspective—Insights from Cerebellar Output

While anatomical and network-level models provide a framework for understanding how stroke affects distributed brain systems, they do not fully capture the physiological mechanisms through which dysfunction emerges in structurally intact regions. In this context, recent work on cerebellar physiology provides critical insights into how alterations in neuronal activity, independent of structural damage, can lead to behavioral impairment. A key contribution comes from recent experimental studies demonstrating that reductions in Purkinje cell firing are sufficient to impair motor function, even in the absence of significant neuronal loss. In particular, Fields and colleagues showed that age-related motor decline is associated with a reduction in Purkinje cell simple spike firing, without major degeneration of cerebellar neurons, and that restoring firing activity, pharmacologically or through circuit modulation, leads to measurable improvements in motor performance [31]. This work provides an informative physiological model illustrating how impaired cerebellar output dynamics may contribute to behavioral dysfunction despite preserved structural integrity, reinforcing broader concepts of cerebellar predictive processing and distributed physiological modulation [32,33]. These findings are consistent with a broader body of electrophysiological literature demonstrating that the cerebellum operates as a timing and prediction system, in which Purkinje cell firing patterns encode error signals and regulate the output of deep cerebellar nuclei [34]. Disruptions in these firing patterns, whether due to altered synaptic input, neuromodulatory changes, or network perturbations, can therefore lead to functional impairment without requiring overt structural damage. Importantly, cerebellar output exerts a powerful influence on distributed brain systems through the deep cerebellar nuclei and cerebello-thalamo-cortical pathways, which modulate activity across motor and associative cortical areas. Experimental work has shown that changes in cerebellar activity can directly alter cortical excitability and behavior, highlighting the cerebellum’s role as a regulator of large-scale network dynamics [35,36]. This reinforces the idea that cerebellar dysfunction can propagate beyond local circuits to affect distributed networks. From this perspective, the findings of Fields et al. introduce a critical conceptual shift: functional suppression of cerebellar output is sufficient to produce behavioral deficits, even when structural integrity is preserved. Although aging-related cerebellar dysfunction and acute ischemic network dysfunction represent biologically distinct processes, these findings provide a useful conceptual analogy suggesting that physiological suppression within intact circuits may contribute to behavioral impairment in distributed systems. Applied to stroke, this framework suggests that network dysfunction does not necessarily require structural disconnection or cell loss, but can arise from physiological downregulation of activity within intact circuits. This is particularly relevant in the context of diaschisis, where remote regions show reduced metabolism, perfusion, and activity despite being structurally preserved.

## 6. Diaschisis as Cerebello-Cortical Loop Dysfunction

The present review does not propose cerebello-cortical dysfunction as a definitive or exclusive mechanism of post-stroke diaschisis. Rather, we present it as a biologically grounded and testable interpretative framework integrating emerging findings from perfusion imaging, connectomics, and cerebellar physiology [5,37]. Building on these considerations, diaschisis can be more precisely conceptualized as a disruption of cerebello-cortical loop function rather than a nonspecific remote effect of focal injury. Within distributed brain networks, this reflects a circuit-level failure in which reduced activity emerges from impaired interactions within polysynaptic loops linking cortex, cerebellum, and thalamus [5,22,23]. Because these circuits are organized as closed systems, perturbation at any node can propagate across the entire loop, leading to widespread dysfunction despite limited structural damage.

Within this framework, classical patterns of diaschisis can be reinterpreted as different expressions of the same process. Crossed cerebellar diaschisis reflects reduced cerebellar input due to disruption of cortico-ponto-cerebellar pathways, whereas thalamic involvement reflects impairment of relay nodes within the same circuits [19,20]. Their frequent co-occurrence supports the view that diaschisis affects integrated circuits rather than isolated regions.

Importantly, this circuit-based interpretation aligns with recent evidence demonstrating that cerebellar-centered networks contribute simultaneously to motor and cognitive functions. In the NeuroImage study by Lee et al. (2026) [38], a structural covariance network centered on lobule VI and Crus I was associated with both physical and cognitive impairment, and its effects were mediated by distributed regions including the thalamus, hippocampus, and orbitofrontal cortex. This finding is particularly informative, as it shows that cerebellar dysfunction does not act locally but propagates through extended cerebello-cerebral systems, linking multiple domains of behavior within a unified network architecture. Such an organization closely mirrors the patterns observed in diaschisis, where remote and multidomain dysfunction emerges from focal perturbations.

The physiological insights discussed in the previous section further reinforce this interpretation. If reduced Purkinje cell firing is sufficient to impair behavior in the absence of structural damage [31], then disruption of input–output dynamics within cerebellar circuits provides a plausible mechanism through which diaschisis can arise. In this context, reduced afferent input to the cerebellum or altered cerebellar output may lead to a state in which the loop remains structurally intact but functionally compromised. This state can be conceptualized as a form of network-level hypoactivity, in which coordinated activity across nodes is diminished, leading to impaired integration of information across motor and cognitive systems.

This framework also provides a coherent explanation for the multidomain nature of post-stroke deficits. Because cerebello-cortical loops are organized in parallel channels supporting different functional domains, disruption of these loops can lead to a combination of motor, cognitive, and affective impairments depending on the circuits involved. Crucially, the same cortical lesion may differentially affect these loops depending on its position within the network, the extent of associated hypoperfusion, and the pre-existing integrity of the system. This offers a mechanistic account of why similar lesions can produce markedly different clinical outcomes.

Taken together, these considerations support a reinterpretation of diaschisis as a manifestation of cerebello-cortical loop dysfunction, in which focal ischemia induces a state of functional disconnection that propagates through structured circuits. In this view, diaschisis is not merely a passive consequence of injury but an active expression of network failure shaped by the architecture and physiology of distributed brain systems. This circuit-based perspective provides a critical link between focal lesions, large-scale brain dysfunction, and outcome variability, and establishes cerebello-cortical loops as a central substrate through which these processes interact. The present framework does not imply that cerebello-cortical systems are uniquely responsible for post-stroke diaschisis. Rather, cerebello-thalamo-cortical loops are emphasized because they constitute one of the best-characterized polysynaptic systems linking motor and cognitive function, are strongly implicated in classical crossed cerebellar diaschisis, and provide a tractable physiological model for studying distributed dysfunction [21,22,23].

Still, methodological Considerations warrant caution in Post-Stroke Functional Imaging. Interpretation of post-stroke functional connectivity remains methodologically challenging. Alterations in BOLD signal may reflect genuine neuronal dysfunction, but can also arise from neurovascular uncoupling, delayed hemodynamic responses, reperfusion-related changes, medication effects, sedation, aging, or cerebral small vessel disease [6,39,40]. Particularly in acute stroke, altered vascular responsiveness may distort estimates of connectivity and complicate interpretation of resting-state fMRI findings. Multimodal approaches integrating perfusion imaging, electrophysiology, and structural connectivity may therefore be necessary to distinguish true network reorganization from physiological or vascular artifacts.

## 7. A Unified Model of Stroke Outcome Variability

The framework developed above supports a shift from lesion-centered to network-based models of stroke outcome, in which clinical variability emerges from the interaction between focal injury and the organization of distributed brain systems. Within this perspective, outcome is not determined solely by infarct characteristics, but by how the lesion perturbs the structure and dynamics of interconnected networks.

We propose a conceptual framework in which stroke outcome variability may emerge from the interaction of three broad factors. First, the topography and severity of the lesion determine the initial disruption of neural activity and connectivity. Second, brain reserve and network vulnerability shape the capacity of the system to compensate for this disruption, with pre-existing atrophy or reduced network efficiency limiting adaptive responses. Third, and critically, diaschisis may contribute to the propagation of dysfunction across distributed circuits, translating focal ischemia into large-scale alterations of brain activity.

Within this model, diaschisis is not simply a byproduct of injury but a central mechanism through which network failure unfolds. By inducing functional suppression in anatomically intact but connected regions, diaschisis reveals the dependence of brain function on coordinated activity within structured circuits. When interpreted in the context of cerebello-cortical loops, this process reflects a breakdown of loop dynamics, in which reduced input and altered output disrupt the integration of motor and cognitive processes across the brain.

This framework provides a mechanistic explanation for why patients with similar lesions may experience markedly different outcomes. Depending on the extent to which critical loops are engaged, and on the resilience of the underlying network architecture, the same focal injury may result in limited, domain-specific deficits or in widespread, multidomain impairment. In this sense, diaschisis may represent one mechanism through which focal lesions exert amplified effects on distributed brain systems, modulating the translation of structural damage into clinical expression.

Importantly, this model also suggests that stroke recovery should be understood as a process of network reorganization and reactivation, rather than solely structural repair. Restoration of function may depend on the normalization of activity within disrupted circuits, including the recovery of cerebello-cortical loop dynamics. This perspective opens new avenues for prognosis and intervention, emphasizing the need to characterize and target network-level dysfunction in addition to lesion characteristics [14,41,42].

However, this framework remains hypothetical and requires prospective multimodal validation integrating lesion characteristics, structural reserve, and longitudinal measures of network dysfunction. Clinically, future studies should determine which dimensions of diaschisis possess independent prognostic value and which imaging modalities are best suited for clinical implementation. Potential candidates include quantitative perfusion asymmetry metrics, functional connectivity measures within cerebello-thalamo-cortical systems, and multimodal indices integrating structural reserve and network dysfunction [6,42]. Practically, the present framework generates several testable predictions: (1) diaschisis burden should independently predict functional outcome beyond lesion volume; (2) combined cerebellar and thalamic diaschisis should associate with multidomain deficits; (3) baseline brain atrophy should interact with diaschisis severity; (4) recovery should correlate with normalization of cerebello-cortical connectivity; and (5) patients with preserved structural reserve but severe functional suppression may preferentially benefit from network-targeted neuromodulation approaches.

Although diaschisis and network disconnection provide a compelling framework for understanding stroke outcome variability, several complementary mechanisms likely contribute to post-stroke dysfunction and recovery. The present framework complements rather than replaces existing models of stroke dysfunction. Traditional lesion-based approaches remain highly informative for acute deficit localization, while structural disconnection models emphasize white matter tract interruption and connectome topology. Similarly, inflammatory and vascular models highlight biological processes that may influence recovery independently of network architecture and pre-existing neurodegenerative changes may also influence network resilience and behavioral outcome [14]. Rather than representing mutually exclusive explanations, these processes likely interact dynamically with diaschisis and distributed network dysfunction.

## 8. Conclusions

Stroke outcomes cannot be fully explained by focal injury alone but reflect the failure of distributed brain systems. In this review, we propose that diaschisis may constitute an important physiological mechanism contributing to distributed network dysfunction after stroke. By integrating perfusion imaging, network neuroscience, and cerebellar physiology, we show that functional suppression within these circuits—rather than structural damage alone—plays a critical role in shaping behavioral outcomes. Within this framework, brain reserve defines network resilience, while diaschisis governs the propagation of dysfunction. This circuit-based perspective provides a mechanistic account of outcome variability and shifts the focus toward network dynamics as both a prognostic marker and a therapeutic target.

## Data Availability

As this study is a review article, no new data were created or analyzed. Therefore, data sharing is not applicable.
